# Micro/Nano Manufacturing

**DOI:** 10.3390/mi8100297

**Published:** 2017-10-02

**Authors:** Guido Tosello

**Affiliations:** Department of Mechanical Engineering, Technical University of Denmark, Niels Koppels Allé, Building 404, 2800 Kgs. Lyngby, Denmark; guto@mek.dtu.dk; Tel.: +45-45-25-4893

Micro- and nano-scale manufacturing has been the subject of an increasing amount of interest and research effort worldwide in both academia and industry over the past 10 years. Traditional lithography-based technology forms the basis of silicon-based micro-electro-mechanical systems (MEMS) manufacturing, but also precision manufacturing technologies have been developed to cover micro-scale dimensions and accuracies. Furthermore, these fundamentally different technology ecosystems are currently combined in order to exploit strengths of both platforms. One example is the use of lithography-based technologies to establish nanostructures that are subsequently transferred to 3D geometries via injection molding.

Manufacturing processes at the micro-scale are the key-enabling technologies to bridge the gap between the nano- and the macro-worlds, to increase the accuracy of micro/nano-precision production technologies, and to integrate different dimensional scales in mass-manufacturing processes. Accordingly, the present Special Issue aims to provide the recent developments in the field of Micro/Nano Manufacturing in terms of production techniques and key enabling technologies that push the boundaries of the state of the art in mass-manufacturing of micro-scale and micro/nano structured components.

The Special Issue consists of 13 original research papers, which cover both fundamental process technologies developments as well as application of those technologies for the fabrication of micro/nano devices. The papers included in the Special Issue addresses research in four main domains of micro/nano manufacturing:

(1) Process developments of micro-scale fabrication methods. Cannella et al. [1] developed a novel tooling system for in-die sintering of micro metal gears; Martinez-López et al. [2] investigated xurography and laser ablation processes for the rapid fabrication of micromixing arrays; Obikawa et al. [3] demonstrated ultrasonic-assisted incremental microforming for the rapid prototyping of thin shell metal pyramids.
(2) Micro/nano manufacturing technologies based on electrochemical processing. Bahr et al. [4] investigated the slurry application and injection system to advance the performance of the Chemical Mechanical Planarization (CMP) process; Blondiaux et al. [5] realized the fabrication of nanostructured steel mold inserts by applying a combination of nanosphere lithography and electrochemical etching; Guo et al. [6] demonstrated the fabrication of mesoscale channels by applying a micro electrochemical machining (µECM) process based on the scanning micro electrochemical flow cell (SMEFC).
(3) Quality control of micro systems and processes. Baruffi et al. [7] demonstrated the application of the replica molding technology for quality control of micro milled surfaces; Cao et al. [8] investigated the effect of profile errors on the surface of micro lenses on laser beam homogenization; Choi et al. [9] presented a novel testing platform to characterize and predict the mechanical damage of miniaturized haptic actuator; Gao et al. [10] modeled the effect of micro manufacturing process variations on the characteristics of a micro machined doubly-clamped beam.
(4) Key enabling technologies for micro production. Aizawa et al. [11] presented the development of low-temperature plasma nitriding for the treatment of stainless miniaturized pipes and nozzles’ internal surfaces; Davoudinejad et al. [12] modeled and simulated the micro end-milling process including the effect of tool run-out; Wilhelmi et al. [13] demonstrated the improvements in multi-stage micro production by handling wire-based linked micro parts.

We wish to thank all authors who submitted their papers to the Special Issue “Micro/Nano Manufacturing”. We would also like to acknowledge all the reviewers whose careful and timely reviews ensured the quality of this Special Issue.
